# A Thermal Infrared Remote Sensing Model for Diagnosing Winter Wheat Water (*Triticum aestivum* L.) Stress by Integrating Angular Effects and Kernel-Driven Models

**DOI:** 10.3390/plants15142201

**Published:** 2026-07-18

**Authors:** Xiaohan Lu, Guoqiang Hu, Xiaofei Yang, Hao Li, Hao Liu, Qi Xu, Yanfu Liu, Daoxu Fan, Zilong Li, Junying Chen, Xin Hui, Maosheng Ge, Zhitao Zhang

**Affiliations:** 1 Key Laboratory of Agricultural Soil and Water Engineering in Arid and Semiarid Areas, Ministry of Education, College of Water Resources and Architectural Engineering, Northwest A&F University, Xianyang 712100, Chinaxiaofei-yang@nwafu.edu.cn (X.Y.); 18306869657@163.com (H.L.); 120330@nwafu.edu.cn (H.L.); husqi@nwafu.edu.cn (Q.X.); liuyanfu@nwafu.edu.cn (Y.L.); 2024050990@nwafu.edu.cn (D.F.); lizilong@nwafu.edu.cn (Z.L.); xinhui@nwafu.edu.cn (X.H.); gmsnongshui@nwafu.edu.cn (M.G.); zhangzhitao@nwafu.edu.cn (Z.Z.); 2 Xinjiang Research Institute of Agriculture in Arid Areas, Northwest A&F University, Urumqi 830091, China; 3 Information Management Division (Network and Educational Technology Center), Northwest A&F University, Xianyang 712100, China; hgq@nwsuaf.edu.cn

**Keywords:** angular effect, canopy temperature, Crop Water Stress Index (CWSI), kernel-driven model, unmanned aerial vehicle (UAV) thermal infrared remote sensing

## Abstract

Canopy temperature (*T*_c_) is an important indicator for characterizing crop water status and serves as the core variable for constructing the Crop Water Stress Index (CWSI). Timely and accurate diagnosis of crop water stress is of great significance for precision irrigation and yield improvement. Owing to its non-contact and high-efficiency characteristics, unmanned aerial vehicle (UAV) remote sensing has become an effective approach for high-spatiotemporal-resolution monitoring of crop water conditions. However, variations in observation geometry can introduce thermal directional effects in canopy temperature, thereby reducing the stability and reliability of CWSI estimation. In this study, multi-angular thermal infrared imagery acquired by a UAV platform was utilized to investigate the directional characteristics of winter wheat canopy temperature. A kernel-driven model was employed to separate the directional components of canopy temperature and retrieve isotropic temperature parameters that more closely represent the actual thermal status of the crop canopy. Based on these temperature parameters, three CWSI models were constructed and evaluated for crop water stress diagnosis. The results demonstrated that (1) winter wheat canopy temperature exhibited pronounced directional characteristics, and the observed temperature generally decreased with increasing relative azimuth angle between the viewing direction and solar incident direction; (2) after angular correction, the isotropic canopy temperature simulated by the kernel-driven model showed an improved correlation with soil moisture content at a depth of 30 cm (R^2^ = 0.54); and (3) when angular-corrected canopy temperature was used as the input variable for different CWSI models, the sensitivity of all models to crop water variation was substantially enhanced, resulting in improved discrimination among different irrigation treatments. Among the evaluated approaches, the empirical CWSI model achieved the best performance in diagnosing crop water stress variations (R^2^ = 0.73, RMSE = 1.59%). These findings provide a theoretical basis for UAV-based thermal infrared remote sensing of crop water status and offer technical support for precision irrigation management.

## 1. Introduction

Water scarcity has become a major constraint on the sustainable development of agriculture in arid and semi-arid regions. Improving agricultural water use efficiency is therefore essential for ensuring food security and promoting the development of modern agriculture [1]. Timely and accurate assessment of crop water status is a critical prerequisite for precision irrigation and efficient farmland water resource management [2]. Canopy temperature is an important parameter reflecting the processes of energy and water exchange within the soil–plant–atmosphere continuum (SPAC) [3,4], and it can effectively indicate crop transpiration and water stress conditions. Under water stress, stomatal closure suppresses transpiration cooling, resulting in an increase in canopy temperature. Consequently, crop water stress monitoring based on canopy temperature has a clear physiological basis. Unmanned aerial vehicle (UAV)-based thermal infrared remote sensing technology enables rapid and non-contact acquisition of crop canopy temperature information, providing important technical support for monitoring crop water status in agricultural fields [5,6,7]. However, the accuracy of UAV-derived canopy temperature may be influenced by both the thermal characteristics of lightweight uncooled thermal infrared sensors and observation geometry, highlighting the need to better understand and correct directional effects in thermal observations.

Changes in crop canopy temperature are jointly influenced by external environmental conditions as well as crop phenotypic characteristics and physiological metabolic activities [8,9,10]. Under conditions of insufficient soil water supply, crop stomata close to reduce transpiration water loss, resulting in decreased latent heat dissipation and increased sensible heat flux, which consequently elevates canopy temperature. This forms the physiological basis for using canopy temperature as an indicator of crop water stress [11]. In 1963, Tanner first proposed the use of canopy temperature to diagnose crop water status [12]. Subsequent studies demonstrated that the canopy air temperature difference (*T*_c_ − *T*_a_) is closely associated with crop transpiration and can effectively reflect crop water stress conditions; however, its stability is relatively poor because it is strongly affected by meteorological factors [3,13]. To improve the applicability of temperature-based indicators, Idso et al. normalized the canopy air temperature difference and proposed the empirical Crop Water Stress Index (CWSI) model [13], while Jackson et al. further developed a theoretical CWSI model based on energy balance theory [3], thereby strengthening its physical basis. Subsequent studies proposed empirical-theoretical hybrid models that combine the simplicity of empirical parameterization with the physical basis of theoretical models, aiming to improve the applicability and robustness of CWSI under complex environmental conditions. Consequently, CWSI has become one of the most widely used thermal indicators for crop water status monitoring [14]. Nevertheless, regardless of the CWSI formulation adopted, its reliability fundamentally depends on the accuracy and consistency of canopy temperature retrieval.

With the development of UAV-based thermal infrared remote sensing technology, CWSI has become capable of monitoring crop water status over large agricultural areas. However, the accuracy of CWSI estimation is highly dependent on canopy temperature inputs. Therefore, obtaining more accurate canopy temperature measurements under complex agricultural backgrounds has become critical for improving the characterization capability of CWSI and the reliability of crop water stress diagnosis [15,16,17]. Crop canopies possess complex three-dimensional structures [18]. Factors such as leaf inclination angle, canopy roughness, and shadow background can result in pronounced thermal directional effects in thermal radiation [19,20]. Under different sun–target–sensor geometries, the apparent canopy temperature acquired by thermal infrared sensors cannot fully represent the actual thermal state of crops [21,22,23]. Previous studies have shown that canopy temperature differences under varying observation angles can reach several degrees Celsius [19], and may even exceed the temperature variations caused by water stress itself, thereby leading to the misinterpretation of observation geometry differences as crop water stress differences [24]. Kimes and Kirchner (1983) were the first to systematically demonstrate the significant angular dependence of canopy temperature observations [25]. Since then, extensive studies have been conducted on the directional characteristics of thermal radiation, and various methods for describing and correcting thermal directional effects have been proposed. Among these approaches, kernel-driven models have been widely adopted for quantifying and correcting thermal directional effects because they provide a physically interpretable framework with relatively few parameters and can effectively describe the influence of canopy structure and viewing geometry on thermal anisotropy [26,27,28,29]. The Li–Strahler–Friedl–Ross–Li (LSF-RL) kernel-driven model proposed by Cao et al. (2021) can retrieve isotropic temperature parameters from multi-angle thermal infrared observations, thereby separating the thermal directional effects of observation geometry on canopy temperature and providing a methodological basis for obtaining temperatures closer to the actual crop thermal state [30,31,32]. However, most existing studies have primarily focused on characterizing or correcting thermal directional anisotropy itself, whereas much less attention has been paid to how observation angle effects propagate into different CWSI formulations and ultimately influence the accuracy of crop water stress assessment [29,31]. Furthermore, the integration of kernel-driven thermal anisotropy correction with different CWSI models for crop water stress diagnosis has rarely been systematically evaluated, limiting the practical application of multi-angle thermal infrared remote sensing in precision irrigation [16,33]. Therefore, it remains necessary to clarify whether correcting thermal directional effects can consistently improve the performance of different CWSI formulations and enhance the reliability of UAV-based crop water stress monitoring.

To address these knowledge gaps, this study used winter wheat as the study crop and employed multi-angular UAV thermal infrared observations to investigate the directional variation patterns of canopy temperature. A kernel-driven model was employed to retrieve isotropic canopy temperature, allowing a systematic comparison of different CWSI formulations before and after thermal directional correction. Using soil moisture content as an external reference variable for water availability, this study quantitatively evaluated the effects of viewing geometry on canopy temperature and CWSI estimates from the perspective of the soil water supply–crop response relationship. In addition, the potential application of isotropic canopy temperature for crop water stress monitoring was further assessed. The results of this study are expected to provide both a theoretical basis and technical support for monitoring crop water status using UAV thermal infrared remote sensing.

## 2. Results

### 2.1. Directional Variation Patterns in Canopy Temperature Under Different Viewing Angles

**Figure 1:**
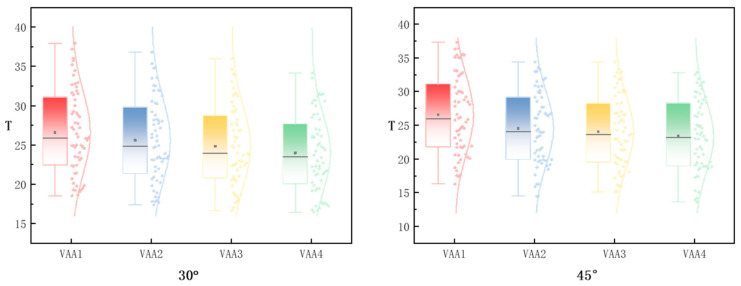
Distribution of canopy temperature retrieved from different viewing angles. Note: VAA1–VAA4 represent four observation azimuths, of which VAA1 represents the hotspot direction; 0° represents the vertical observation direction.

Multi-angular thermal infrared images acquired by an unmanned aerial vehicle (UAV) under clear-sky conditions during the 2025 winter wheat growing season were processed to retrieve canopy temperature, and the resulting directional variation patterns under different irrigation treatments are presented in Figure 1. From the perspective of viewing geometry, canopy temperature generally exhibited a decreasing trend with increasing relative azimuth angle between the sensor viewing direction and the solar incident direction. When the viewing direction approached the solar incident direction (i.e., the hotspot direction), shadows within the canopy were largely obscured, while sunlit leaves and stems dominated the sensor field of view. Consequently, the contribution of high-temperature components to the observed thermal radiation increased, resulting in the maximum observed canopy temperature. As the relative azimuth angle increased, shadows within the canopy and between crop rows became progressively exposed. The proportion of low-temperature shaded areas within the sensor field of view therefore increased, leading to an overall decline in canopy temperature.

**Figure 2:**
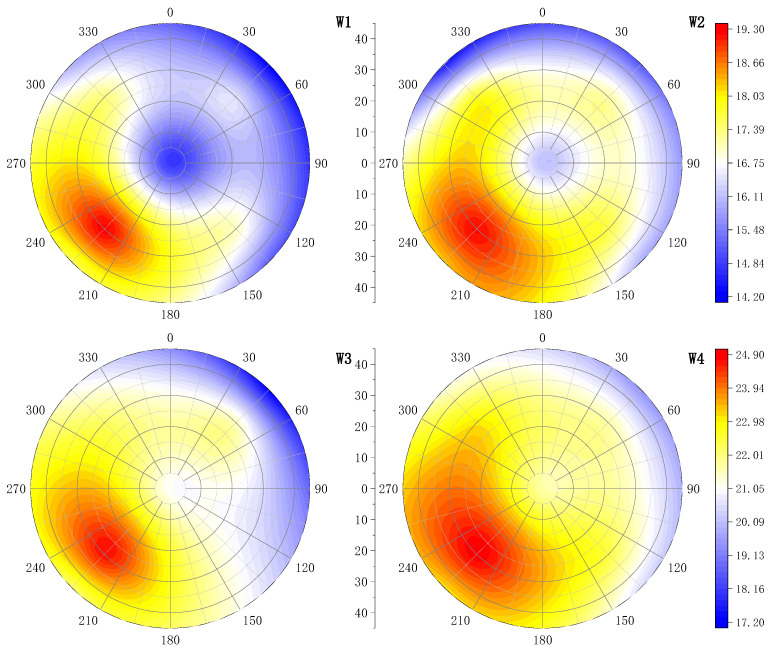
Directional distribution of canopy thermal radiation.

The canopy temperature under different irrigation treatments exhibited pronounced gradient characteristics. As shown in Figure 2, canopy temperature increased progressively with decreasing irrigation level, with the lowest temperature observed under the W1 treatment and the highest temperature observed under the W4 treatment. In addition, a distinct hotspot effect was observed, whereby the maximum canopy temperature occurred in the hotspot viewing direction. However, the intensity of the hotspot effect gradually weakened with decreasing irrigation level. The radiation energy balance of crops is primarily regulated through photosynthesis, transpiration, and longwave radiation exchange. When soil water availability decreases, crop stomata gradually close to reduce transpiration water loss, leading to a substantial decline in evaporative cooling capacity and consequently an increase in overall canopy temperature. The formation of the hotspot effect is jointly controlled by canopy geometric structure and internal temperature heterogeneity and is strongly influenced by viewing geometry. Under severe water stress conditions, leaf wilting and canopy structural degradation reduce the thermal heterogeneity within the canopy and weaken the directional shadowing effect among leaves, thereby diminishing the directional characteristics of thermal radiation. These results demonstrate that canopy temperature exhibits pronounced thermal anisotropy, indicating that viewing geometry should be explicitly considered when using UAV thermal infrared observations to monitor crop water status.

### 2.2. Response of Canopy Temperature to Water Stress Under Different Viewing Angles

**Figure 3:**
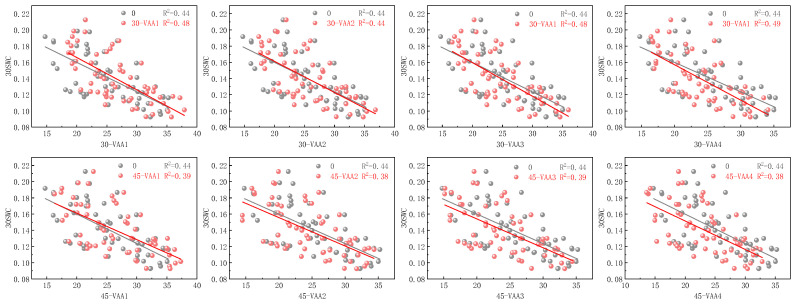
Relationship between multi-angle canopy temperature and soil moisture content at 30 cm depth.

Linear regression analysis was performed to quantify the relationship between canopy temperature under different viewing geometries and soil moisture content (SMC) at a depth of 30 cm, thereby evaluating the sensitivity of canopy temperature to crop water stress. The results revealed pronounced differences in the sensitivity of canopy temperature to water stress among different viewing angles (Figure 3). Using the canopy temperature derived from the conventional nadir observation angle (VZA = 0°) as the reference, the coefficient of determination between canopy temperature and SMC at a depth of 30 cm was 0.44. Under a viewing zenith angle (VZA) of 30°, the coefficient of determinations obtained from the four viewing azimuth angles (VAA1–VAA4) were 0.48, 0.44, 0.48, and 0.49, respectively, all equal to or higher than the reference value, indicating a consistently stronger relationship with soil moisture than that obtained from the nadir observation. These results suggest that moderately oblique observations enhance the sensitivity of canopy temperature to water stress induced by soil water availability. However, when the viewing zenith angle increased to 45°, the correlation coefficients for all viewing azimuth directions decreased to below 0.40, which was lower than that under the nadir condition, indicating that excessively large viewing angles weaken the response of canopy temperature to water stress. These results indicate that increasing the viewing zenith angle does not necessarily improve the sensitivity of canopy temperature to water stress. Instead, an intermediate viewing angle (approximately 30° in this study) provides a more favorable balance between observing sunlit canopy components and minimizing background interference.

Under the same viewing zenith angle, the four viewing azimuth directions (VAA1-VAA4) generally exhibited consistent response patterns to water stress, although directional differences were still observed. At VZA = 30°, the hotspot direction (30°-VAA1) showed slightly higher correlation than the dark-side direction (30°-VAA4), indicating stronger sensitivity to water stress. In contrast, an opposite tendency was observed under the 45° condition, where the 45°-VAA1 direction yielded the highest correlation coefficient (0.39), while the 45°-VAA4 direction exhibited the lowest value.

These directional differences are mainly attributed to variations in canopy structure and viewing geometry. At moderate viewing zenith angles (VZA ≈ 30°), the sensor field of view captures both upper canopy leaves and part of the internal canopy structure, thereby enhancing the representation of transpiration cooling effects in the thermal infrared signal and increasing the sensitivity of canopy temperature to water stress induced by soil water availability. In contrast, at larger viewing zenith angles (VZA ≈ 45°), the proportions of non-photosynthetic organs, shaded components, and soil background within the field of view increase substantially. As a result, the thermal signal is increasingly influenced by a mixture of shadow-induced cooling and soil thermal emission, thereby weakening the relationship between canopy temperature and water stress. Overall, canopy temperature exhibits distinct responses to water stress under different viewing geometries. These results demonstrate that viewing geometry should be considered when constructing temperature-based water stress indicators such as the CWSI. This observation is consistent with the thermal radiation transfer mechanism of crop canopies, in which the relative contributions of sunlit leaves, shaded leaves, and soil background vary with viewing geometry.

### 2.3. Kernel-Driven Model for Multi-Angular Temperature Simulation and Water Stress Response Characteristics

**Figure 4:**
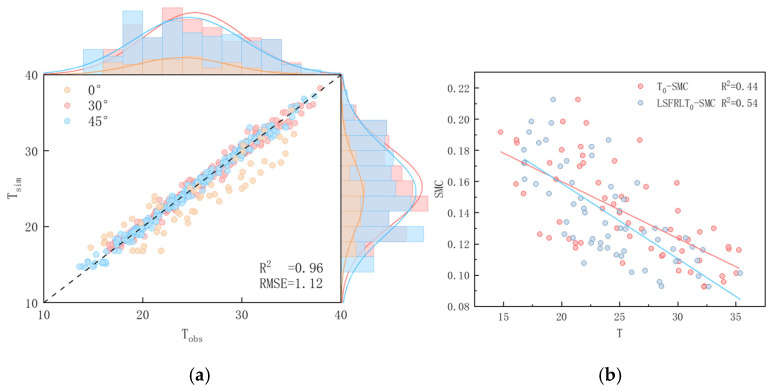
(**a**) Correlation between the original canopy temperature extracted from UAV imagery and the simulated temperature generated using the kernel-driven model. (**b**) Correlation between the original orthorectified temperature and the orthorectified temperature simulated using the LSF-RL model with soil moisture content. Different colors indicate different observation angles. The dashed line in (**a**) represents the 1:1 line. The red and blue regression lines in (**b**) represent the fitted relationships for the original and LSF-RL simulated temperatures, respectively.

Due to the pronounced directional characteristics of canopy temperature, the sensitivity of temperature information acquired under different viewing angles to variations in water status varies considerably. Therefore, multi-angular observations were used to construct a kernel-driven model to correct temperature anisotropy, enabling the simulation and decomposition of canopy thermal directional effects. The results (Figure 4a) showed a high consistency between simulated and observed canopy temperatures, with a coefficient of determination (R^2^) of 0.96. The overall regression was established using observations collected from all sampling dates and viewing configurations. This indicates that the LSF-RL kernel-driven model can effectively characterize the variation in winter wheat canopy thermal radiation under different viewing geometries, demonstrating strong multi-angular temperature simulation capability.

From the model fitting results (Table 1), the LSF-RL model preserved the general hotspot pattern, with relatively higher canopy air temperature differences still observed in the hotspot viewing direction. Compared with the original observations, however, the magnitude of directional temperature differences among viewing geometries was reduced after kernel-driven correction. This indicates that the kernel-driven model primarily reduced thermal anisotropy associated with viewing geometry rather than altering the overall spatial pattern of canopy temperature. In addition, statistical results in Table 1 shows that after kernel-driven modeling, temperature differences among different viewing zenith angles were reduced. This suggests that the model can attenuate thermal directional variation patterns induced by differences in viewing geometry, thereby producing smoother and more stable multi-angular temperature patterns. Meanwhile, the relative temperature differences among irrigation treatments were preserved after correction, indicating that the kernel-driven model mainly reduced directional effects while maintaining the canopy temperature response to soil water availability. Accordingly, the ranking of canopy temperatures among the four irrigation treatments (W1–W4) remained unchanged after kernel-driven correction.

**Figure 5:**
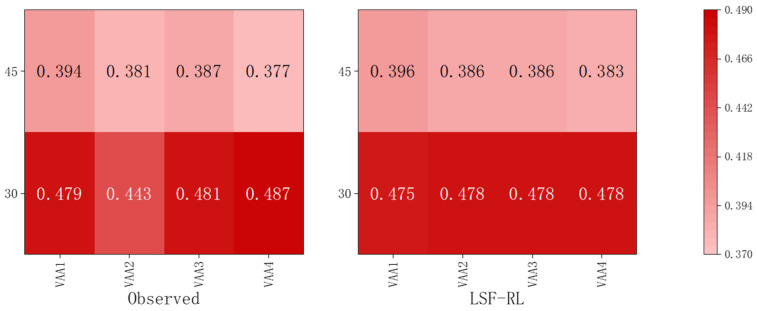
Correlation between original and simulated canopy temperature and soil moisture content at 30 cm depth.

Regression analysis was conducted to investigate the relationship between canopy temperature simulated by the kernel-driven model under eight inclined viewing geometries and winter wheat soil moisture content at 30 cm depth. A correlation heatmap was generated (Figure 5). The results indicate that lighter colors represent weaker correlations, while darker colors indicate stronger correlations. From the vertical direction, the correlation between canopy temperature and 30 cm soil moisture content generally decreased with increasing viewing zenith angle. From the horizontal direction, the variation patterns across different viewing azimuth angles (VAA1–VAA4) were largely consistent, suggesting that the influence of azimuth angle on the temperature–soil moisture relationship is relatively limited. Compared with the original observations, the kernel-driven model consistently increased the coefficients of determination across different viewing geometries while reducing the variability among viewing directions. This indicates that the kernel-driven model can partially mitigate the influence of viewing geometry on the temperature-soil moisture relationship.

Among the three kernel coefficients, the isotropic coefficient ${(f}_{\mathrm{iso}})$ represents the canopy temperature component after removing directional effects and therefore provides an angularly independent characterization of canopy thermal conditions. The orthographic canopy temperature reconstructed from the isotropic term was further correlated with 30 cm soil moisture content (Figure 4b), yielding a coefficient of determination (R^2^) of 0.54, which is notably higher than that obtained under single-view observations. These results demonstrate that incorporating multi-angular observations and separating directional components via the kernel-driven model can provide a more physically representative characterization of canopy thermal conditions, thereby improving the capability of thermal infrared remote sensing for diagnosing crop water stress.

### 2.4. Changes in CWSI After Considering Angular Effects

Canopy temperature extracted from original UAV thermal infrared images and the simulated nadir-view canopy temperature derived from the isotropic coefficient of the kernel-driven model were used to calculate CWSI under different water treatment conditions, respectively. The mean statistical results are shown in Table 2. Overall, CWSI values from all models increased with increasing water stress, indicating their ability to characterize winter wheat water status.

Without considering angular effects, all three CWSI indices showed an increasing trend with increasing water stress severity. Specifically, CWSIt increased from 0.24 to 0.63 under W1–W4 treatments, CWSIe increased from 0.09 to 0.76, and CWSIh increased from 0.45 to 0.96. All three indices were able to effectively distinguish different water treatments. After angular correction using kernel-driven model-derived nadir temperature, CWSI values generally decreased across all water treatments. CWSIt decreased from 0.24 to 0.17 under W1, with a reduction of approximately 29%. CWSIe decreased from 0.76 to 0.66 under W4, and CWSIh decreased from 0.96 to 0.87 under W4. Overall, all indices showed varying degrees of reduction after angular correction, indicating that the directional characteristics of canopy temperature affect CWSI estimation. This reduction can be attributed to the removal of directional temperature overestimation caused by hotspot observations. After reducing thermal anisotropy, canopy temperature more closely represents the actual thermal status of the canopy, thereby improving the physical consistency between CWSI and crop water status.

**Figure 6:**
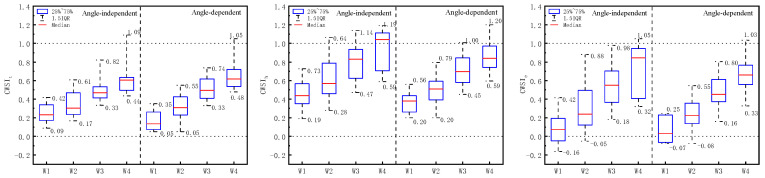
Variation in CWSI considering the angular effect.

The distribution characteristics of CWSI before and after considering angular effects are shown in Figure 6. The results indicate that the median values of the theoretical and empirical models are relatively close, while the hybrid model exhibits an overall higher median value. In terms of distribution range, the theoretical model shows the smallest dispersion, with most values concentrated between 0 and 1. The empirical model presents the widest distribution range, varying from −0.16 to 1.05, while the hybrid model lies between the other two. After angular correction, the distribution ranges of all three CWSI models were reduced, and the separation among different water treatments was further improved. This indicates that correcting thermal directional effects reduces the variability introduced by viewing geometry, resulting in more consistent CWSI estimates across different observation conditions. Among the three models, CWSIe shows the largest variation range. This suggests that the empirical CWSI is more sensitive to variations in canopy temperature and therefore benefits more from thermal directional correction. After angular correction, its values range from 0.07 to 0.66, indicating high sensitivity to water stress variations.

### 2.5. Response of CWSI Based on the Isotropic Coefficient of the Kernel-Driven Model to Water Stress

**Figure 7:**
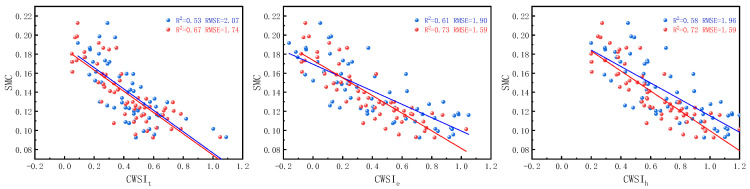
Estimation of soil moisture content using UAV thermal infrared CWSI. The red and blue regression lines represent the relationships obtained using the original CWSI and the LSF-RL simulated CWSI, respectively.

During nadir-view imaging by traditional UAV-mounted thermal infrared cameras, the hottest canopy tops directly illuminated by sunlight and facing the sensor are easily captured, leading to overestimation of canopy temperature and consequently affecting the accuracy of CWSI estimation. Therefore, multi-angular information was introduced in this study, and the isotropic coefficient derived from the kernel-driven model, which minimizes thermal directional effects and better represents the actual canopy thermal condition, was used to calculate different CWSI models. Because the isotropic coefficient represents the canopy temperature component independent of viewing geometry, it provides a more physically consistent input for CWSI calculation than the original single-view canopy temperature. To evaluate their capability for characterizing crop water stress, linear regression models were established using the least squares method, with soil moisture content at 30 cm depth as the reference indicator of soil water availability. Although soil moisture cannot fully represent the physiological response of crops to water stress, it provides an objective indicator of root-zone water availability under the controlled irrigation treatments adopted in this study. The performance of different CWSI models in characterizing crop water stress was then assessed. The results are shown in Figure 7.

The results indicate that all CWSI models were negatively correlated with soil moisture content. After considering angular effects, all three CWSI models showed improved relationships with soil moisture content, with R^2^ increasing by 0.10–0.14 and RMSE decreasing by 0.31–0.37%. However, differences were observed among the models. Overall, for both the original nadir-view canopy temperature data and the simulated data, under the experimental conditions of this study, the empirical CWSI model achieved the highest correlation with soil moisture content, followed by the hybrid model, whereas the theoretical model showed relatively lower performance. After angular correction, the R^2^ values of both the empirical and hybrid models exceeded 0.70, whereas the theoretical model remained below 0.70. The hybrid model showed the largest improvement, with R^2^ increasing by 0.14 and RMSE decreasing by 0.37%. Before considering angular effects, the empirical model achieved an R^2^ of 0.61 and an RMSE of 1.90% for soil moisture estimation. After angular correction, the R^2^ increased to 0.73, while the RMSE decreased to 1.59%. Regardless of whether angular effects were considered, the empirical model consistently showed the strongest response to soil moisture variation. These results demonstrate that correcting thermal directional effects can enhance the consistency between CWSI and soil water availability under the experimental conditions of this study.

To further evaluate model robustness, leave-one-date-out cross-validation was performed. As shown in Table 3, the cross-validated performance remained close to the original results for all angular-corrected CWSI models. The empirical model achieved a cross-validated R^2^ of 0.69 and an RMSE of 1.68%. Paired *t*-tests further confirmed that the reduction in prediction errors after angular correction was significant (*p* < 0.05), indicating that the improved performance was not caused by substantial overfitting.

**Figure 8:**
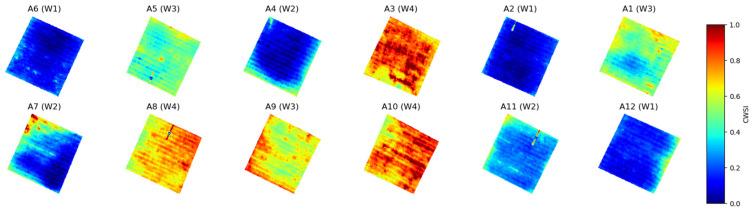
CWSI inversion map derived from UAV thermal infrared imagery.

Based on the best-performing UAV thermal infrared model for crop water monitoring, namely the empirical CWSI model considering angular effects, a UAV thermal infrared remote sensing inversion map of crop water stress was generated, as shown in Figure 8. The results show that CWSI values range from 0 to 1, with colors changing from red to blue representing increasing CWSI values. Distinct spatial differences are observed among different water treatments, and the overall distribution pattern is consistent with the experimental irrigation design. Within the same treatment, the spatial distribution of CWSI is relatively uniform, although certain local variations still exist, mainly due to differences in illumination conditions and irrigation non-uniformity. The resulting spatial distribution generally agreed with the irrigation treatment layout, indicating the potential of the proposed method for field-scale visualization of crop water status. It should be noted that the inversion map was generated under controlled experimental conditions involving a single winter wheat cultivar and one growing season. Therefore, further validation across different cultivars, environments, and growing seasons is still required before broader application.

## 3. Discussion

### 3.1. Effects of Crop Water Status on the Thermal Directional Characteristics of the Canopy

Crop canopy thermal infrared radiation exhibits pronounced directional characteristics [30,31,34], and crop water status can further influence these directional variation patterns. The results of this study demonstrated that winter wheat canopy temperature showed clear water-gradient characteristics under different irrigation treatments, mainly because crop transpiration plays a critical role in regulating canopy energy balance. In addition to affecting the overall canopy temperature level, reduced water supply gradually induces stomatal closure and weakens the transpiration cooling effect [11], thereby increasing canopy temperature and altering the directional characteristics of canopy thermal radiation. The results further showed that under relatively sufficient water conditions (W1 and W2), canopy thermal radiation exhibited a pronounced hotspot effect. However, with increasing water stress, the hotspot effect gradually weakened. This phenomenon is likely associated with the influence of water stress on canopy structure. Under well-watered conditions, leaves maintain relatively high erectness and a more intact canopy structure, resulting in stronger mutual shading effects among leaves and consequently producing a distinct hotspot phenomenon under specific viewing directions [35,36,37]. In contrast, under severe water stress conditions, physiological responses such as leaf wilting and curling alter the canopy structure and reduce the spatial occlusion among leaves, thereby weakening the directional characteristics of canopy thermal radiation and limiting its ability to accurately reflect the actual crop water status. Although leaf wilting and canopy structural changes provide a reasonable explanation for the observed directional characteristics, quantitative measurements of canopy structural parameters, such as leaf area index (LAI) and leaf inclination angle, were not available in this study. Future work should incorporate these measurements to further verify the underlying mechanisms.

### 3.2. Effects of the Kernel-Driven Model on Multi-Angular Canopy Temperature

Canopy thermal radiation exhibits pronounced anisotropic characteristics, and temperature information acquired from different viewing angles often differs considerably, thereby limiting the application of thermal infrared remote sensing in crop water monitoring to some extent. Therefore, a kernel-driven model was employed to simulate and decompose multi-angular canopy temperature observations in order to reduce the influence of observation geometry on canopy temperature. The results demonstrated that the LSF-RL kernel-driven model effectively captured the variation patterns of winter wheat canopy temperature under different viewing geometries, with good agreement between simulated and measured temperatures [30,38]. Compared with the original observed temperatures, the temperature differences among viewing angles were reduced after kernel-driven model correction. The model was able to smooth fluctuations among multi-angular temperature observations to a certain extent, thereby minimizing the influence of observation geometry on temperature information. Although radiometric correction was performed using the DJI Thermal SDK, the retrieved canopy temperature may still be influenced by the characteristics of the uncooled thermal infrared sensor. Nevertheless, the strong agreement between the measured and simulated temperatures suggests that the proposed correction framework effectively reduced directional effects under the experimental conditions of this study. In addition, the isotropic component derived from the kernel-driven model represented the canopy temperature component independent of viewing-angle effects. Compared with temperature observations from a single viewing angle, the simulated nadir temperature calculated from the isotropic component showed a stronger correlation with soil moisture content. These findings indicate that incorporating multi-angular observations and applying directional decomposition of canopy temperature using the kernel-driven model can provide more stable and representative temperature parameters, thereby improving the reliability of thermal infrared remote sensing for monitoring crop water status.

### 3.3. Effects of Angular Effects on the Performance of UAV Thermal Infrared Remote Sensing Crop Water Stress Indices

After considering angular effects, the ability of different CWSI models to characterize crop water status was improved (Figure 6 and Figure 7). Canopy temperature retrieved from UAV-based thermal infrared remote sensing is significantly influenced by observation geometry, and substantial differences in thermal radiation signals can occur under different viewing angles [39,40,41]. When the viewing direction approaches the solar illumination direction, a larger proportion of sunlit leaves is included within the sensor field of view, while shaded regions inside the canopy are obscured, resulting in relatively higher observed temperatures. As the relative azimuth angle between the viewing direction and solar illumination direction increases, shaded and low-temperature regions within the canopy gradually become exposed, leading to a decrease in observed canopy temperature [42]. Since CWSI is calculated based on the canopy–air temperature difference, such directional variation patterns in canopy temperature directly affect CWSI estimation and consequently influence its capability to detect crop water stress.

By incorporating multi-angular observations and applying a kernel-driven model to decompose the directional characteristics of canopy temperature, the influence of observation geometry on temperature signals can be effectively reduced, thereby obtaining more stable canopy temperature parameters. Since CWSI is fundamentally constructed based on canopy thermal status and transpiration-driven water consumption processes [43], reducing directional errors enables canopy temperature to more accurately reflect crop transpiration cooling conditions, thereby enhancing the capability of CWSI to characterize water stress. Consequently, the corrected canopy temperature better represents the intrinsic thermal status of the crop canopy rather than temperature variations introduced by observation geometry, resulting in a more reliable characterization of crop water status by CWSI. However, different CWSI models exhibited varying responses after considering angular effects. Empirical models are primarily established based on statistical relationships between canopy–air temperature differences and environmental temperature [13,44], making them more sensitive to canopy temperature variations. Consequently, introducing angular correction can substantially improve their model stability. In contrast, theoretical models are derived from energy balance equations and characterize crop water stress through the physical relationships between transpiration and environmental meteorological factors. These models inherently account for variations associated with canopy energy exchange processes to some extent [3,4,45], and therefore are less affected by angular effects. Hybrid models combine the characteristics of empirical and theoretical approaches, enabling them to effectively capture canopy temperature variation trends while maintaining computational stability. As a result, their overall performance after angular correction was intermediate between the other two model types. A comprehensive comparison among different models indicated that empirical models exhibited higher stability and stronger water-stress responsiveness after angular correction. These findings are generally consistent with previous studies regarding the application of CWSI in crop water monitoring, while further demonstrating that observation angle effects are an important factor influencing CWSI accuracy. This issue, however, has received relatively limited attention in previous UAV thermal infrared studies. In this study, the complete multi-angular UAV acquisition required approximately 19 min under stable meteorological conditions, indicating that short-term environmental variation had a limited influence on the observed directional differences. Nevertheless, simplified angular sampling strategies should be investigated in future studies to improve operational efficiency for large-scale applications.

### 3.4. Limitations and Future Perspectives

The present findings should be interpreted within the experimental conditions of this study, which involved one winter wheat cultivar, one site, and one growing season. Nevertheless, measurements from 12 plots across five sampling dates provided 60 plot-date observations, and leave-one-date-out cross-validation showed that the angular-corrected CWSI models retained performance close to that obtained using the complete dataset. This result suggests a limited risk of substantial overfitting. Further multi-site and multi-year validation is required to assess model transferability across cultivars and environmental conditions.

Root-zone soil moisture at 20–30 cm depth was selected as the external reference because it represents soil water availability within an active rooting layer and is less affected by short-term surface evaporation than shallow soil moisture. It therefore provides a relatively stable and objective reference under controlled irrigation treatments. Future studies could combine soil moisture with stomatal conductance, leaf water potential, transpiration rate, leaf area index, and leaf inclination angle to further clarify the physiological and structural mechanisms underlying crop water stress and canopy thermal directionality.

Canopy temperature was retrieved from DJI Zenmuse H30T imagery using the sensor-specific radiometric correction workflow implemented in the DJI Thermal SDK, which incorporates imaging distance, surface emissivity, atmospheric humidity, and reflected ambient temperature. This procedure provided a consistent basis for comparisons among viewing geometries and irrigation treatments. Field blackbody targets could be introduced in future experiments to provide additional independent validation of absolute temperature accuracy.

The complete multi-angular acquisition required approximately 19 min under stable weather conditions, limiting the influence of short-term environmental variation on the comparison among viewing geometries. For larger fields and routine applications, future work should investigate whether comparable correction performance can be achieved using fewer viewing directions.

Overall, angular correction provided a more representative canopy temperature input and improved the capability of CWSI to characterize crop water status. The proposed framework therefore provides a basis for subsequent multi-site validation and operational optimization of UAV thermal infrared monitoring.

## 4. Materials and Methods

### 4.1. Study Area

The study area was located at the Key Laboratory of Agricultural Soil and Water Engineering in Arid and Semiarid Areas of the Ministry of Education, Yangling Demonstration Zone, Shaanxi Province, China (108°4′20″ E, 34°17′42.17″ N; altitude: 525 m). The region is characterized by a temperate semi-arid to semi-humid continental monsoon climate, with precipitation mainly concentrated from July to September and a mean annual precipitation of approximately 640 mm. The soil type within the 0–60 cm layer of the experimental field was medium loam. The average field capacity and wilting coefficient were 26% and 8.6% (volumetric water content), respectively. The soil bulk density was 1.44 g·cm^−3^, and the soil pH was 8.1. Groundwater in the study area is deeply buried, and its upward recharge contribution to the root zone can therefore be considered negligible.

### 4.2. Experimental Design

**Figure 9:**
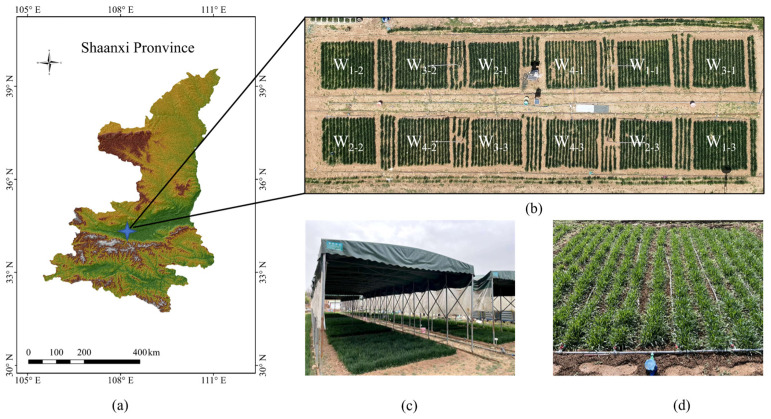
(**a**) Geographical location of the experimental site. (**b**) Layout plan of experimental plots. (**c**) Movable rain shelter device. (**d**) Schematic diagram of a drip irrigation system.

During the winter wheat growing season, regulated irrigation and water-control treatments were implemented using a drip irrigation system. Four irrigation levels were established in this study, including full irrigation (W1, 95% of field capacity), mild water stress (W2, 80% of field capacity), moderate water stress (W3, 65% of field capacity), and severe water stress (W4, 50% of field capacity). Each treatment included three replicates, resulting in a total of 12 experimental plots. The plots with different water treatments were arranged randomly (Figure 9b). Each experimental plot measured 4 m by 4 m, with 1.5 m buffer zones established between adjacent plots. All plots were equipped with water meters and drip irrigation pipes to ensure uniform and quantitative irrigation, thereby maintaining relatively consistent wheat growth conditions within each plot. To strictly maintain the designed water treatment conditions, a movable rain shelter was installed over the entire experimental area to eliminate the influence of natural precipitation [46]. Winter wheat was manually sown in mid-October 2024 using the cultivar Xinong 9112. The row spacing was 25 cm, with a planting density of 200 kg·hm^−2^. A compound fertilizer at a rate of 40 kg·mu^−1^ was applied as basal fertilizer, and 30 g of seeds were sown per row. During the experimental period, strict irrigation control was maintained. Soil moisture measurements conducted one day before irrigation were used to calculate the required irrigation amount, whereas the soil moisture data used for model evaluation were collected during each UAV measurement campaign [47,48].

### 4.3. Experimental Data Acquisition

#### 4.3.1. UAV Thermal Infrared Data Acquisition

**Figure 10:**
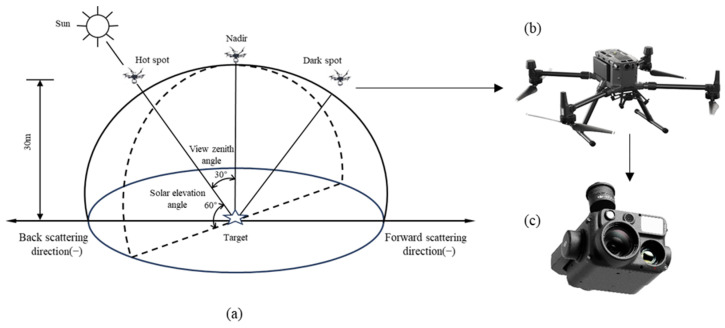
(**a**) Multi-angle UAV flight schematic. (**b**) DJI Matrice 300 RTK platform. (**c**) DJI Zenmuse H30T multi-sensor payload.

Remote sensing data acquisition was conducted under clear-sky conditions with low wind speed and stable illumination. Thermal infrared images were collected using a DJI Matrice 300 RTK platform (Figure 10b) equipped with a DJI Zenmuse H30T multi-sensor payload (SZ DJI Technology Co., Ltd., Shenzhen, China) (Figure 10c). The thermal infrared sensor employed an uncooled VOx microbolometer with a spatial resolution of 1280 × 1024 pixels, a noise equivalent temperature difference (NETD) of ≤50 mK, an equivalent focal length of 52 mm, and a field of view of approximately 45.2° × 37.8°. The UAV flight altitude was set to 30 m with a flight speed of 1 m·s^−1^. The forward and side overlap ratios were configured at 85% and 80%, respectively. The viewing azimuth angle (VAA) was controlled by adjusting the flight path direction, while the viewing zenith angle (VZA) was regulated through the gimbal pitch angle [49]. For each flight mission, one nadir observation and four oblique viewing configurations combining different UAV viewing azimuth angles (VAA) with two viewing zenith angles (VZA) were acquired, resulting in a total of nine flight routes (Figure 10a). The flights were conducted when the solar zenith angle was approximately 45° under clear-sky and low-wind conditions. The complete acquisition of the nine flight routes required approximately 19–20 min, including approximately 3 min for the nadir flight and about 8 min for each oblique observation set (four viewing azimuth angles at a fixed viewing zenith angle). As all flight routes were completed consecutively within a single acquisition window, temporal variations in solar zenith angle, solar radiation, ambient temperature, and other environmental conditions were expected to have limited influence relative to the directional differences in canopy temperature (Table 4) [31].

#### 4.3.2. Ground Data Collection

Ground measurements were conducted on 18 March, 24 March, 3 April, 9 April, and 20 April 2025. Soil moisture content (SMC) data and meteorological observations were simultaneously collected during each campaign, resulting in a total of 60 samples.

Meteorological data were continuously recorded using a portable weather station, including air temperature, relative humidity, wind speed, and net solar radiation. The weather station recorded measurements at 2 min intervals, and the average values within ±10 min of the UAV image acquisition time were used for subsequent analysis.

Soil moisture content was determined using the gravimetric oven-drying method. Soil samples were collected from the 20–30 cm soil layer at the center of each experimental plot using a soil auger. The wet soil mass was first measured, after which the samples were dried in an oven at 105 °C until a constant weight was achieved. Soil gravimetric water content was calculated based on the ratio of water mass loss to dry soil mass. Subsequently, volumetric soil moisture content was obtained by combining the gravimetric water content with soil bulk density [50,51].

### 4.4. Data Processing

#### 4.4.1. Thermal Infrared Data Preprocessing

Raw R-JPEG thermal images acquired by the DJI Zenmuse H30T were radiometrically calibrated using the DJI Thermal SDK v1.7 (SZ DJI Technology Co., Ltd., Shenzhen, China) to generate RAW-format thermal data. The DJI Zenmuse H30T employs an uncooled VOx microbolometer detector, which may be affected by sensor temperature drift, atmospheric conditions, and environmental thermal fluctuations. Parameters, including imaging distance, target surface emissivity, air humidity, and ambient reflected temperature, were incorporated to correct for atmospheric attenuation and environmental reflected radiation, thereby reducing the uncertainty associated with thermal measurements. Subsequently, plot-scale temperature data under different viewing zenith angle (VZA) and viewing azimuth angle (VAA) conditions were obtained and exported as TIFF files. A statistical threshold method implemented in Python 3.12.7 (Python Software Foundation, Wilmington, DE, USA) was applied to remove abnormal temperature pixels within each plot. The average value of the remaining valid pixels was calculated as the representative canopy temperature for each experimental plot. The 1280 × 1024 thermal imagery allowed the individual 4 m by 4 m experimental plots to be clearly delineated. Plot-level canopy temperature was calculated using valid pixels within each plot boundary after abnormal pixels were removed. However, oblique observations may increase the projected pixel footprint and plot-boundary mixing, and these effects could not be completely eliminated in the present study.

#### 4.4.2. Kernel-Driven Model

To quantitatively characterize the directional thermal radiation properties of the winter wheat canopy, a kernel-driven thermal directional model based on the Ross–Li kernel framework, namely the LSF-RL model, was employed to simulate multi-angular thermal infrared observations. The LSF-RL model was selected because it combines the Ross–Thick volumetric scattering kernel and the Li–Sparse geometric-optical kernel, providing a balance between physical interpretability and computational efficiency for describing thermal directional anisotropy over crop canopies. Using a kernel-function decomposition approach, canopy temperature was represented as a linear combination of isotropic, volumetric scattering, and geometric-optical components. The model effectively characterizes the influence of canopy structure and sun–sensor viewing geometry on thermal radiation anisotropy [30,52]. The model can be expressed as follows:

(1) $$T\left(\theta_{v},\Delta \phi \right)=f_{\mathrm{iso}}+f_{\mathrm{vol}}\;K_{\mathrm{vol}}\left(\theta_{v},\theta_{s},\Delta \phi \right)+f_{\mathrm{geo}}\;K_{\mathrm{geo}}\left(\theta_{v},\theta_{s},\Delta \phi \right)$$

where $T\left(\theta_{v},\Delta \phi \right)$ represents the canopy temperature observed at viewing zenith angle $\theta_{v}$ and relative azimuth angle Δϕ; $\theta_{s}$ denotes the solar zenith angle; $f_{\mathrm{iso}}\;f_{\mathrm{vol}}\;\mathrm{and}\;f_{\mathrm{geo}}$ are the weighting coefficients of the isotropic, volumetric scattering, and geometric-optical components, respectively. The kernel coefficients were estimated independently for each experimental plot and observation date by fitting the nine observed canopy temperatures from different viewing configurations to Equation (1) using ordinary least-squares linear regression, resulting in one set of coefficients (${\;f}_{\mathrm{iso}}\;f_{\mathrm{vol}}\;\mathrm{and}\;f_{\mathrm{geo}})$ for each observation date.

(1) Ross–Thick Volumetric Scattering Kernel

The volumetric scattering kernel describes the canopy volume-scattering effect caused by the random spatial distribution of leaves [26], and is expressed as follows:

(2) $$K_{\mathrm{vol}}=\frac{\left(\frac{\pi }{2}-\xi \right)\cos\xi +\sin\xi }{\cos\theta_{s}+\cos\theta_{v}}-\frac{\pi }{4},$$

where

(3) $$\cos\xi =\cos\theta_{s}\cos\theta_{v}+\sin\theta_{s}\sin\theta_{v}\cos\Delta \phi ,$$

and ξ is the phase angle between the solar and viewing directions.

(2) Li–Sparse Geometric-Optical Kernel

The geometric-optical kernel characterizes thermal directional effects caused by canopy structural occlusion, leaf overlap, and shadow variations [53], and is expressed as follows:

(4) $$K_{\mathrm{geo}}=O\left(\theta_{s},\theta_{v},\Delta \phi \right)-\sec\theta_{s}-\sec\theta_{v}+\frac{1}{2}\left(1+\cos\xi \right)\sec\theta_{s}\sec\theta_{v},$$

where $O\left(\theta_{s},\theta_{v},\Delta \phi \right)$ denotes the geometric overlap function, which describes the overlap degree of canopy structural projections in the solar and viewing directions.

By fitting the kernel-driven model to the multi-angular canopy temperatures, the three kernel coefficients $f_{\mathrm{iso}}f_{\mathrm{vol}}\;\mathrm{and}\;f_{\mathrm{geo}}$ can be obtained. Among them, $f_{\mathrm{iso}}$ primarily reflects the overall canopy thermal condition and is highly sensitive to crop water status. The coefficient $f_{\mathrm{vol}}$ characterizes the volumetric scattering effect induced by leaf spatial distribution, whereas $f_{\mathrm{geo}}$ represents the geometric directional characteristics associated with canopy structural occlusion and shadow fraction variations. These kernel coefficients can further be used to analyze the directional variation pattern characteristics of winter wheat canopy thermal radiation under different water conditions. The retrieved isotropic coefficient $(f_{\mathrm{iso}}$) was used to reconstruct the isotropic canopy temperature, whereas $\;f_{\mathrm{vol}}\;\mathrm{and}\;f_{\mathrm{geo}}$ were used to quantify the contributions of volumetric scattering and geometric-optical effects to canopy thermal anisotropy.

### 4.5. Crop Water Stress Index

The Crop Water Stress Index (CWSI) was used to characterize the degree of crop water stress, and its general form is expressed as follows:

(5) $$CWSI=\frac{(T_{c}-T_{a})-(T_{c}-T_{a}{)}_{LL}}{(T_{c}-T_{a}{)}_{UL}-(T_{c}-T_{a}{)}_{LL}},$$

where $T_{c}-T_{a}$ is the canopy air temperature difference; $(T_{c}-T_{a}{)}_{LL}$ is the lower baseline of the canopy–to–air temperature difference under non–water–stressed conditions; and $(T_{c}-T_{a}{)}_{UL}$ is the upper baseline under fully stressed conditions. A CWSI value of 0 indicates that the crop is under sufficient water supply without water stress, whereas a value of 1 indicates severe water stress. According to different methods for determining the upper and lower baselines, CWSI models can be categorized into theoretical, empirical, and hybrid approaches.

#### 4.5.1. Theoretical Crop Water Stress Index (${CWSI}_{t}$)

Jackson et al. proposed a theoretical CWSI model in 1981 based on the energy balance equation and the Penman–Monteith equation. The canopy air temperature difference can be expressed as follows:

(6) $$\left(T_{c}-T_{a}\right)=\frac{r_{a}\left(R_{n}-G\right)}{\rho c_{P}}\cdot \frac{\gamma \left(l+\frac{r_{c}}{r_{A}}\right)}{\Delta +\gamma \left(1+\frac{r_{c}}{r_{a}}\right)}-\frac{VPD}{\Delta +\gamma \left(1+\frac{r_{c}}{r_{a}}\right)},$$

where $r_{a}$ is the aerodynamic resistance (s/m); $R_{n}$ is the net radiation (W/m^2^); G is the soil heat flux (W/m^2^); ρ is the air density (kg/m^3^); $c_{P}$ is the specific heat capacity of air at constant pressure (J/(kg·K)); γ is the psychrometric constant (Pa/K); $r_{c}$ is the canopy resistance (s/m); Δ is the slope of the saturation vapor pressure curve (Pa/K); and VPD is the vapor pressure deficit (Pa).

When the canopy resistance approaches infinity ($r_{c}\to \infty$), the crop is assumed to be under complete water stress, and the canopy air temperature difference reaches its maximum value. Under this condition, the upper baseline can be expressed as follows [3]:

(7) $$(T_{c}-T_{a}{)}_{UL}=\frac{r_{a}\left(R_{n}-G\right)}{\rho c_{P}},$$

When the canopy resistance equals the potential canopy resistance ($r_{c}=r_{Cp}$), the crop is considered to be under well-watered conditions, and the canopy air temperature difference reaches its minimum value. Jackson et al. assumed that under sufficient soil moisture conditions the crop canopy could be regarded as a free water surface, i.e., $r_{cp}=0\;s/m$. Therefore, the lower baseline can be calculated as follows:

(8) $$(T_{c}-T_{a}{)}_{LL}=(T_{c}-T_{a}{)}_{UL}\frac{\gamma }{\Delta +\gamma }-\frac{VPD}{\Delta +\gamma }=\frac{r_{a}\left(R_{n}-G\right)}{\rho c_{P}}\cdot \frac{\gamma }{\Delta +\gamma }-\frac{VPD}{\Delta +\gamma },$$

The aerodynamic resistance $r_{a}$ was calculated as follows:

(9) $$r_{A}=\left\{\begin{array}{c} \frac{4.72{\left[\frac{\ln\left(z-d\right)}{z_{0}}\right]}^{2}}{\Delta +\gamma },\;\;WS\leq 2\;m/s\\ \frac{{\left[\frac{\ln\left(z-d\right)}{z_{0}}\right]}^{2}}{k^{2}WS},\;\;WS>2\;m/s \end{array}\right.,$$

where z is the reference height (m); d  is the zero-plane displacement height (m), calculated as 0.63h, h is the crop height (m); $z_{0}$ is the roughness length (m), calculated as 0.13h; WS is the wind speed at the reference height (m/s), with the reference height set to 2 m; and k is the von Kármán constant, which was set to 0.4.

The vapor pressure deficit (VPD) was calculated as follows:

(10) $$VPD=0.6108exp\left(\frac{17.27\times T_{a}}{T_{a}+237.7}\right)\cdot \frac{100-RH}{100},$$

where $T_{a}$ is the air temperature (°C), and RH is the relative humidity (%).

#### 4.5.2. Empirical Crop Water Stress Index (${CWSI}_{e}$)

Idso et al. reported that under clear-sky conditions, the canopy air temperature difference in well-watered crops exhibited a linear relationship with VPD. Therefore, the lower baseline of the empirical CWSI model can be expressed as follows:

(11) $$(T_{c}-T_{a}{)}_{LL}=a\cdot VPD+b,$$

where a and b are regression coefficients determined under well–watered conditions.

The upper baseline of the empirical CWSI model was defined as the maximum observed canopy air temperature difference under severe water stress conditions:

(12) $$(T_{c}-T_{a}{)}_{UL}=max\left(T_{c}-T_{a}\right),$$

#### 4.5.3. Hybrid Crop Water Stress Index (${CWSI}_{h}$)

Ekinzog et al. indicated that both empirical and theoretical CWSI models have certain limitations. The lower baseline of the empirical model is highly affected by environmental conditions, whereas the theoretical model requires numerous meteorological and canopy parameters. Therefore, the hybrid model adopts the empirically determined upper baseline and the theoretically calculated lower baseline to improve model stability while simplifying the calculation process. The upper baseline of the hybrid model was calculated using Equation (12), whereas the lower baseline was derived from Equation (8) as follows:

(13) $$(T_{c}-T_{a}{)}_{LL}=(T_{c}-T_{a}{)}_{UL}\frac{\gamma }{\Delta +\gamma }-\frac{VPD}{\Delta +\gamma }=max(T_{c}-T_{a})\frac{\gamma }{\Delta +\gamma }-\frac{VPD}{\Delta +\gamma },$$

### 4.6. Modeling Methods and Accuracy Evaluation

Simple linear regression was employed to characterize the relationship between each Crop Water Stress Index (CWSI) formulation and crop water status by minimizing the residual sum of squares. To evaluate the capability of different CWSI formulations in representing crop water conditions, soil moisture content (SMC) was selected as the reference indicator, and separate regression models were established between SMC and each CWSI formulation. The coefficient of determination $R^{2}$ and root mean square error (RMSE) were used as evaluation metrics. A higher $R^{2}$ value approaching 1 and a lower RMSE value approaching 0 indicate better model performance and monitoring accuracy. To further assess the robustness of the regression models, leave-one-date-out cross-validation was performed. Specifically, data from four sampling dates were used for model calibration, while the remaining sampling date was used for validation, and the procedure was repeated until each sampling date had served once as the validation dataset. The predictions from all held-out dates were pooled, and the cross-validated R^2^ and RMSE were calculated from the combined out-of-fold predictions. In addition, paired *t*-tests were conducted to evaluate whether the prediction errors of the angular-corrected CWSI models were significantly lower than those of the original models, with statistical significance determined at *p* < 0.05. The corresponding equations are expressed as follows:

(14) R2=1−∑i=1n(yi^−y¯)2∑i=1n(yi−y¯)2,

(15) RMSE=∑i=1n(yi−yi^)2n,

where $y_{i}$ represents the measured SMC value, yi^ represents the predicted SMC value, $\overset{¯}{y}$ denotes the mean SMC value, and n is the sample size. In this study, SMC was introduced as an environmental reference variable to evaluate the effectiveness of CWSI in characterizing crop water stress, thereby enabling the linkage between soil water supply conditions and crop physiological responses.

## 5. Conclusions

In this study, canopy temperature and meteorological data of winter wheat in 2025 were collected using a UAV thermal infrared imager and a meteorological station. Three CWSI models were calculated with and without considering angular effects, and their abilities to characterize crop water status were evaluated. The results showed that:

(1) Winter wheat canopy temperature exhibited significant directional characteristics, with substantial temperature differences observed under different viewing angles. As the relative azimuth angle between the viewing direction and solar illumination direction increased, canopy shadows gradually became exposed, resulting in an overall decrease in observed canopy temperature. In addition, decreasing water status led to higher canopy temperature and a weakened hotspot effect.
(2) The LSF-RL kernel-driven model effectively simulated the directional characteristics of winter wheat canopy thermal radiation and obtained isotropic canopy temperature by separating directional components. The isotropic temperature showed a coefficient of determination (R^2^) of 0.54 with soil moisture content at a depth of 30 cm which was higher than that of temperatures obtained from a single viewing angle, indicating that it could more accurately characterize crop water status.
(3) After considering observation angle effects, the response capability of different CWSI models to water variation was significantly enhanced by calculating CWSI using isotropic canopy temperature derived from the kernel-driven model, and the discrimination among different water treatments was improved. Among the different models, the empirical model achieved the best performance (R^2^ = 0.73, RMSE = 1.59%), indicating its higher reliability in characterizing crop water stress and responding to soil moisture variation.

## Figures and Tables

**Table 1:** Comparison of mean canopy air temperature difference (*T*_c_ − *T*_a_) retrieved from different viewing angles. Note: T_obs_: observed canopy temperature; *T*_sim_: simulated canopy temperature; *T*_a_: air temperature. (*T*_obs_ − *T*_a_) and (*T*_sim_ − *T*_a_) represent canopy air temperature difference (°C).

(T − T_a_, °C)	Water Treatment (W)	0°	30°				45°			
		0	VAA1	VAA2	VAA3	VAA4	VAA1	VAA2	VAA3	VAA4
T_obs_ − T_a_	W1	2.78	4.11	3.24	2.49	1.75	4.15	2.58	1.84	1.15
	W2	5.51	6.34	5.70	4.78	3.83	6.56	4.43	4.26	3.53
	W3	8.54	9.58	8.48	7.73	6.99	9.34	7.46	6.88	6.28
	W4	11.24	12.08	10.79	10.08	9.13	11.95	9.48	8.91	8.28
T_sim_ − T_a_	W1	1.65	4.03	2.68	2.68	2.20	4.35	1.97	1.97	1.44
	W2	3.83	6.35	4.94	4.94	4.43	6.70	4.21	4.21	3.66
	W3	7.02	9.41	7.96	7.96	7.44	9.55	6.99	6.99	6.43
	W4	9.13	11.98	10.24	10.24	9.62	12.13	9.06	9.06	8.38

**Table 2:** Comparison of mean CWSI values considering angular effects.

Index	Angle-Independent				Angle-Dependent			
	W1	W2	W3	W4	W1	W2	W3	W4
${CWSI}_{t}$	0.24	0.36	0.50	0.63	0.17	0.31	0.51	0.65
${CWSI}_{e}$	0.09	0.30	0.55	0.76	0.07	0.24	0.49	0.66
${CWSI}_{h}$	0.45	0.61	0.80	0.96	0.36	0.51	0.73	0.87

**Table 3:** Robustness evaluation of the angular-corrected CWSI models. Note: Full-dataset R^2^ and RMSE were calculated using the complete dataset, whereas cross-validated R^2^ and RMSE were obtained by leave-one-date-out cross-validation. The *p*-values were derived from paired *t*-tests comparing model prediction errors before and after angular correction.

Model	Full-Dataset R^2^	Cross-Validated R^2^	Full-Dataset RMSE	Cross-Validated RMSE	Paired *t*-Test (*p*)
${CWSI}_{t}$	0.67	0.59	1.74	1.93	0.007
${CWSI}_{e}$	0.73	0.69	1.59	1.68	0.014
${CWSI}_{h}$	0.72	0.68	1.59	1.71	0.012

**Table 4:** Observation time and solar geometry.

Date	18 March	24 March	3 April	9 April	20 April
Time (SZA = 45°)	14:55	15:06	15:20	15:27	15:37
SAA (°)	225°	230°	238°	243°	250°

Note: SZA, solar zenith angle; SAA, solar azimuth angle.

## Data Availability

The original contributions presented in this study are included in the article. Further inquiries can be directed to the corresponding author.
